# Effect of a multidisciplinary lifestyle intervention on body composition in people with osteoarthritis: Secondary analysis of the “Plants for Joints” randomized controlled trial

**DOI:** 10.1016/j.ocarto.2024.100524

**Published:** 2024-10-01

**Authors:** C.A. Wagenaar, W. Walrabenstein, C.S. de Jonge, M. Bisschops, M. van der Leeden, M. van der Esch, P.J.M. Weijs, M.A. Troelstra, M.A. Korteweg, A.J. Nederveen, D. van Schaardenburg

**Affiliations:** a Reade Center for Rheumatology and Rehabilitation, Amsterdam, the Netherlands; b Amsterdam UMC Location University of Amsterdam, Department of Clinical Immunology and Rheumatology, Meibergdreef 9, Amsterdam, the Netherlands; c Amsterdam Rheumatology and Immunology Center, Amsterdam, the Netherlands; d Amsterdam UMC Location University of Amsterdam, Department of Radiology and Nuclear Medicine, Meibergdreef 9, Amsterdam, the Netherlands; e Amsterdam Gastroenterology Endocrinology Metabolism Research Institute, Amsterdam, the Netherlands; f Amsterdam UMC Location Vrije Universiteit Amsterdam, Department of Rehabilitation Medicine, Boelelaan 1117, Amsterdam, the Netherlands; g Amsterdam Movement Sciences Research Institute, Amsterdam, the Netherlands; h Center of Expertise Urban Vitality, Amsterdam University of Applied Sciences, Faculty of Health, Amsterdam, the Netherlands; i Department of Nutrition and Dietetics, Center of Expertise Urban Vitality, Amsterdam University of Applied Sciences, Amsterdam, the Netherlands; j Amsterdam UMC Location Vrije Universiteit Amsterdam, Department of Nutrition and Dietetics, Boelelaan 1117, Amsterdam, the Netherlands

**Keywords:** Osteoarthritis, Diet, Lifestyle intervention, Metabolic syndrome, Body composition, Magnetic resonance imaging

## Abstract

**Objective:**

The Plants for Joints (PFJ) intervention significantly improved pain, stiffness, and physical function, and metabolic outcomes, in people with metabolic syndrome-associated osteoarthritis (MSOA). This secondary analysis investigated its effects on body composition.

**Method:**

In the randomized PFJ study, people with MSOA followed a 16-week intervention based on a whole-food plant-based diet, physical activity, and stress management, or usual care. For this secondary analysis, fat mass, muscle mass, and bone mineral density were measured using dual-energy X-ray absorptiometry (DEXA) for all participants. Additionally, in a subgroup (*n* ​= ​32), hepatocellular lipid (HCL) content and composition of visceral adipose tissue (VAT) were measured using magnetic resonance spectroscopy (MRS). An intention-to-treat analysis with a linear-mixed model adjusted for baseline values was used to analyse between-group differences.

**Results:**

Of 66 people randomized, 64 (97%) completed the study. The PFJ group experienced significant weight loss (−5.2 ​kg; 95% CI –6.9, −3.6) compared to controls, primarily from fat mass reduction (−3.9 ​kg; 95% CI –5.3 to −2.5). No significant differences were found in lean mass, muscle strength, or bone mineral density between groups. In the subgroup who underwent MRI scans, the PFJ group had a greater reduction in HCL (−6.5%; 95% CI –9.9, 3.0) compared to controls, with no observed differences in VAT composition.

**Conclusion:**

The PFJ multidisciplinary intervention positively impacted clinical and metabolic outcomes, and appears to significantly reduce body fat, including liver fat, while preserving muscle mass and strength.

## 1 Introduction

The ‘Plants for Joints’ (PFJ) randomized controlled trial investigated the effect of a multidisciplinary lifestyle intervention based on an *ad libitum* (unrestricted calorie intake) whole food plant based diet, physical activity, and stress management in people with metabolic syndrome-associated hip and/or knee osteoarthritis (MSOA) [1]. After the 16-week intervention, MSOA participants had significantly less pain and stiffness, and improved physical function [2]. Participants also showed improved metabolic status, including significant improvements in weight, fat mass, HbA1c, and LDL-cholesterol [2]. These changes in body composition and metabolic health are relevant as excess visceral adipose tissue (VAT), a significant trigger of inflammation, is involved in the pathophysiology of MSOA [[3], [4], [5], [6]]. Outcomes regarding the effects of the PFJ intervention on muscle mass and strength, bone mineral density (BMD), liver fat, and VAT composition were not yet reported.

The assessment of these factors are important as plant-based diets typically contain less protein than animal products [7], with lower protein quality and bioavailability [8], potentially affecting muscle mass. Also, a higher bone fracture risk has been found in vegans compared to omnivores [9]. As a result, potential concerns of plant-based diets on muscle mass and BMD exist. Regarding the fat compartment, excess VAT contributes to insulin resistance and inflammation via liver fat accumulation and by the composition of its fatty acids [3,10,11]. Saturated fatty acids in VAT are associated with insulin resistance and inflammation, while unsaturated fatty acids are protective [12]. VAT fatty acid composition may be reflective of diet and is correlated to dietary intake [13,14].

Therefore, this secondary analysis of the PFJ study aims to determine the effect of the PFJ intervention on body composition, including muscle mass and strength, BMD, liver fat and VAT composition.

## 2 Methods

### 2.1 Design

A 16-week open-label RCT with parallel design was conducted between May 2019 and December 2021 ​at the Reade outpatient clinic for rehabilitation and rheumatology in Amsterdam, The Netherlands. Study visits took place at baseline, 8 and 16 weeks. The Medical Ethical Committee of the Amsterdam University Medical Centers approved the study protocol (EudraCT number NL66649.048.18). Study protocols were prospectively registered (International Clinical Trial Registry Platform numbers NL7800 and NL7801) and published [1]. Participants gave written informed consent. The study was performed in accordance with the Declaration of Helsinki and followed the Consolidated Standards of Reporting Trials (CONSORT) reporting guideline [15].

### 2.2 Study sample

Sample size calculations and exclusion criteria were previously described [1,2]. Randomization was concealed using the digital CASTOR electronic data capture system that allocated participants to the intervention or control group in a 1:1 ratio, with block randomization in block sizes of 2 and 4. Inclusion criteria were ≥18 years, metabolic syndrome according to the National Cholesterol Education Program criteria and knee or hip OA according to the American College of Rheumatology clinical criteria [[16], [17], [18]].

### 2.3 Intervention

Details of the PFJ intervention were previously published [1,2]. Briefly, the intervention consisted of 10 group sessions with 6–12 participants in which theoretical and practical education about a whole-food plant-based diet, physical activity, and sleep and stress management were discussed according to the Dutch nutrition [19] and physical activity guidelines (150 ​min/week moderate intense physical activity and 2 days/week musculoskeletal strengthening activities) [20]. The control group received usual care and was advised not to change their lifestyle habits.

### 2.4 Body composition measurements

Dual-energy X-ray absorptiometry (DEXA) was used to measure total lean mass, total fat mass, appendicular skeletal muscle mass (ASMM, sum of lean mass in arms and legs) and BMD at baseline and at the end trial. DEXA scans were performed by a technician blinded to group allocation on a whole-body scanner (Lunar iDXA enCORE version 17, GE Medical Systems, United States). Body weight and waist circumference were measured by a research dietician.

### 2.5 Magnetic resonance spectroscopy measurements

All MSOA participants were asked during inclusion whether they gave additional consent to undergo two MRI scans. The subgroup of participants who consented underwent a ^1^H MRS scanning protocol at baseline and after 16 weeks in supine position on a 3T MRI scanner (Ingenia, Philips Healthcare, Best, The Netherlands) using a posterior coil located in the table and an anterior torso-coil covering the abdominal region. MRS data were collected in liver tissue and VAT, in accordance with a previous study [21,22]. Spectra were recorded with a multi-echo stimulated-echo acquisition mode single-voxel localization sequence centered on the water frequency (repetition time 3500 ​ms; spectral width 2000 hz).

Hepatocellular lipid (HCL) content was measured using a single voxel (20 ​× ​20 ​× ​20 ​mm^3^) positioned in the right hepatic lobe (Fig. 2), avoiding major blood vessels, bile ducts and liver margins. Five spectra were acquired (echo times 10, 15, 20, 25, 30 ​ms) during a breath hold at end-expiration. Fatty acid composition of VAT was measured using a single voxel (15 ​× ​25 ​× ​25 ​mm^3^) positioned in the VAT retroperitoneal under the right kidney. Acquisition was performed during breath hold at end-expiration with an echo time of 9.5 ​ms. HCL and VAT spectral data were fitted in the time domain using a nonlinear least-squares algorithm (AMARES) in jMRUI v4.0 and Matlab R2021a (Mathworks, Natick, MA, USA). Spectra were excluded if they were not interpretable, for example, due to absence of a fat peak in the liver. HCL was measured by calculating the proton density fat fraction following the procedure described by Runge et al. [21]. MRS derived proton density fat fraction is a non-invasive, accurate, and reproducible method to assess liver steatosis and non-alcoholic fatty liver disease (NAFLD) [21,22].

To measure VAT composition, ^1^H-MR spectra were manually phased using jMRUI and referenced to the methylene resonance at 1.3 ​ppm. AMARES was used to estimate lipid signal amplitudes for 10 peaks according to pre-defined peak assignments and Gaussian line shapes [23,24]. To establish a prior knowledge file that captures resonance peaks while limiting the fitting residual, theoretical amplitudes were combined with trial and error of different line widths, phases, and frequencies (Supplementary Table 1) [23,24]. To control for factors impacting peak amplitude during acquisition (e.g. spectral line width, signal-to-noise ratio, chemical shift artifacts) a ratio was calculated with another peak of the same spectra influenced in the same way. For VAT, amplitude ratios were calculated for the (poly)unsaturated fat peaks (olefin (5.3 ​ppm; unsaturated fat), α-olefin (2.03 ​ppm; unsaturated fat), and/or diacyl (2.77 ​ppm; polyunsaturated fat)) divided by the saturated fat (methylene (1.3 ​ppm)) peak. To ensure complete measurement of the peaks of interest, the amplitude of the α-carboxyl (2.25 ​ppm) peak was summed with the α-olefin peak, and the glycerol peak (5.21 ​ppm) with the olefin peak as these peaks partially overlapped.

### 2.6 Other measurements

Hand grip strength was assessed using a calibrated dynamometer (Jamar Hydraulic Hand Dynamometer) at baseline and 16 weeks, with the mean value of three attempts from the dominant hand. Insulin, alanine aminotransferase (ALAT), aspartate aminotransferase, and estimated glomerular filtration rate were measured from blood samples. Adverse events were recorded and previously described [2].

### 2.7 Statistical analysis

Intention-to-treat analyses were conducted to asses between group differences at the end of the intervention using linear mixed models for variables with three time points and linear regression models for variables with two data points, adjusting for baseline values. Analyses were performed for both the entire population, MSOA subgroups (knee, hip, or both knee and hip MSOA), and the MRS subgroup (i.e. participants who underwent an MRI scan). In cases where model assumptions were not met, outcomes were analyzed after log transformation. Additional analyses were performed adjusting for sex, age, and BMI. Differences between groups at baseline or within groups were analyzed using two- or one-sample t-tests when normally distributed or Wilcoxon-Rank tests when skewed. All analyses were conducted using R version 4.2.2 (2022-10-31), with significance set at *p* ​< ​0.05.

## 3 Results

### 3.1 Participant characteristics

**Fig. 1:**
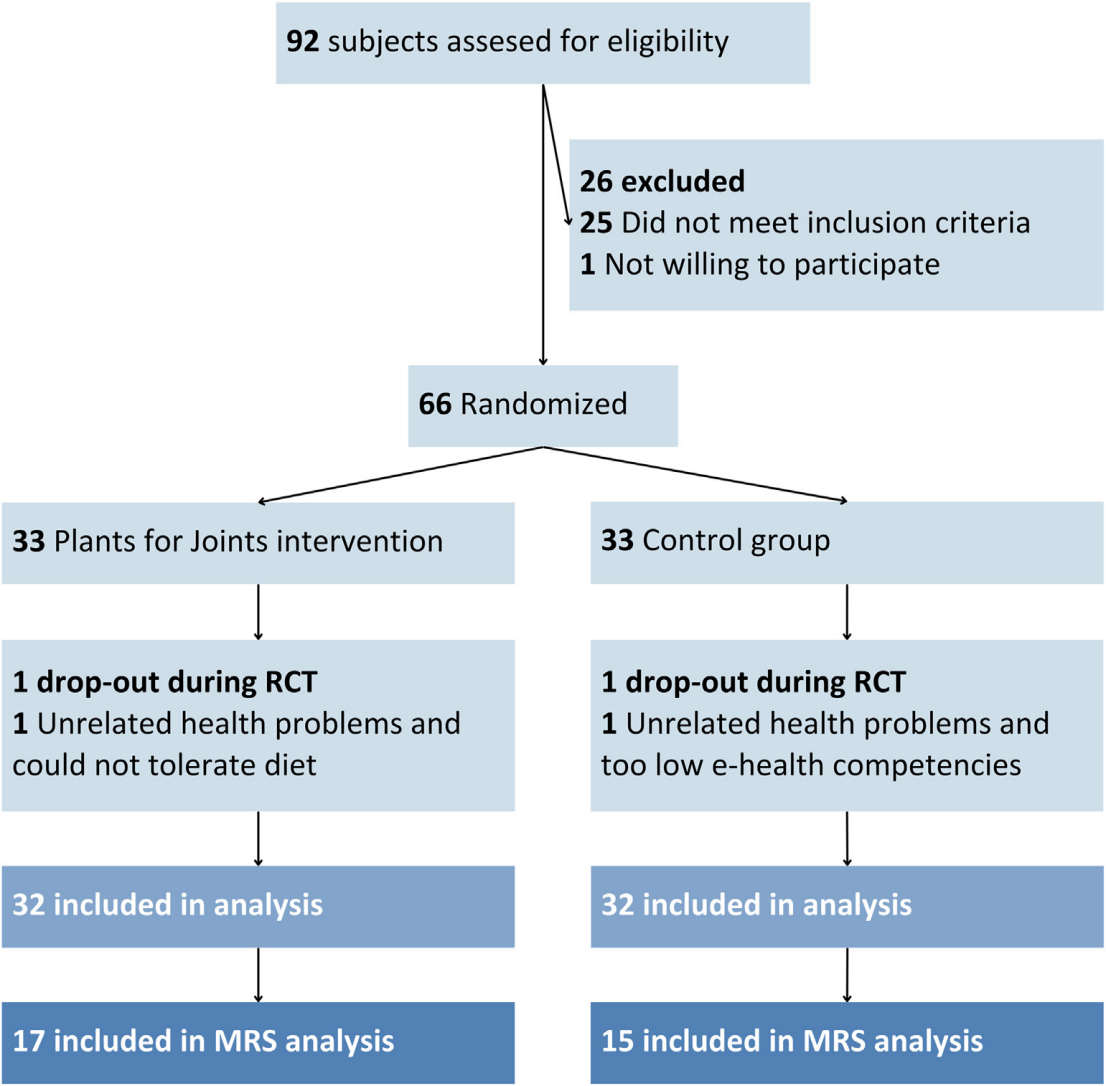
CONSORT flow diagram in the “Plants for Joints” Trial. RCT ​= ​randomized controlled trial, MRS ​= ​magnetic resonance spectroscopy.

Of the 66 people randomized, 64 (97%) were included in the analyses, of which 32 took part in the MRS study (Fig. 1). Two participants dropped-out shortly after randomization: one from the intervention group due to health issues unrelated to the intervention and diet intolerance, and one from the control group dropped out due to health problems and low e-health skills. At baseline, participants had an average (SD) age of 63 (6) years, a mean BMI of 33 (5) kg/m,^2^ and 84% were female. All participants fulfilled the clinical criteria for MSOA and most (*n* ​= ​28 (88%) in PFJ group; *n* ​= ​29 (91%) in control group) the American College of Rheumatology radiological criteria for hip or knee OA.

**Fig. 2:**
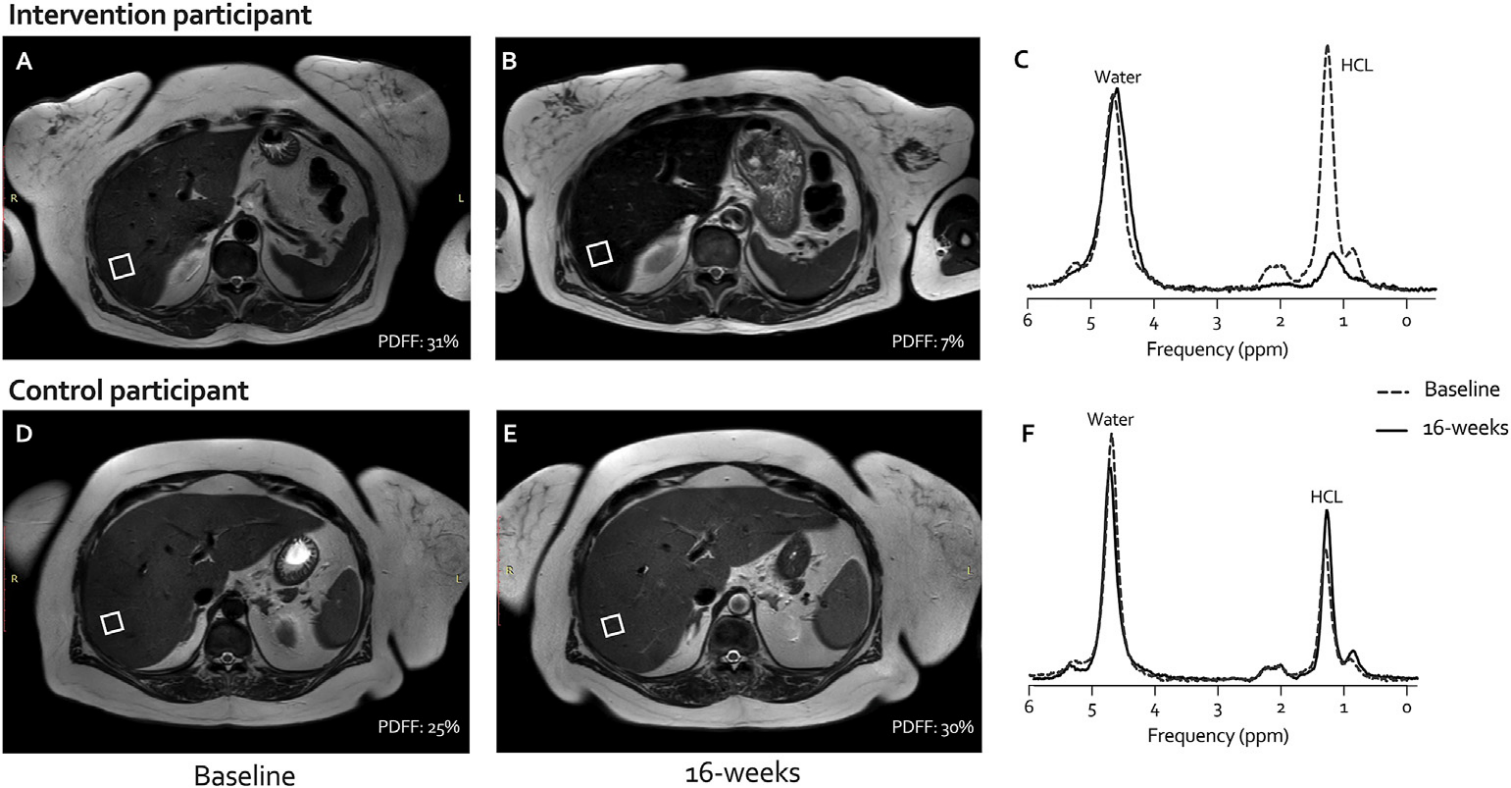
Axial T2W liver MRIs of an intervention participant before (A) and after (B) following the 16-week Plants for Joints intervention and a control group participant at baseline (D) and after 16-weeks (E), along with corresponding acquired MR spectra (C, F). The white boxes show the corresponding MRS voxel placement for hepatocellular fat (HCL) quantification (PDFF) derived from the acquired MRS spectra (C, F) at baseline (dotted line) and end-intervention measurements (solid line).

Twenty-five participants had only knee MSOA (*n* ​= ​9 intervention, *n* ​= ​16 control), 12 only hip MSOA (*n* ​= ​7 intervention, *n* ​= ​5 control), and 25 both knee and hip MSOA (*n* ​= ​16 intervention, *n* ​= ​11 control). At baseline participants with only knee MSOA had greater weight, fatmass, and lean mass (98.6 ​kg (SD 17.0), 43.0 (11.1)), and 52.3 (10.2), respectively) as compared to the whole MSOA cohort (95.0 ​kg (15.9), 41.9 (10.6), and 49.5 (8.7)), those with both knee and hip MSOA (93.6 ​kg (15.4), 41.9 (10.7), and 47.6 (7.1)), and those with only hip MSOA (90.6 ​kg (14.2), 39.8 (9.8), and 47.7 (7.5)).

### 3.2 Body composition

The PFJ group lost significantly more body weight (−5.2 ​kg), fat mass (−3.9 ​kg), fat percentage (−2.1), and waist circumference (−6 ​cm) compared to controls after the intervention (Table 1). The PFJ group showed a trend towards reduced lean mass (−0.7 ​kg 95% CI –1.5 to 0.1) and appendicular skeletal muscle mass (−0.5 ​kg 95% CI –1.0 to 0.1) compared to the control group. There was no difference in bone mineral density (−0.01 ​g/cm^2^ 95% CI –0.03 to 0.01) or grip strength (1 ​kg 95% CI –2 to 4) between groups after 16-weeks (Table 1).

**Table 1:** Outcomes for all metabolic syndrome-associated osteoarthritis participants.

Characteristic	Plants for Joints group (*n* ​= ​32)			Control group (*n* ​= ​32)			Between group	*p*-value
	Baseline	8 weeks	16 weeks	Baseline	8 weeks	16 weeks	difference (95 ​% CI)	
**Body composition**								
Weight, kg	94.6 (17.5)	91.6 (16.2)	88.2 (16.0)	95.3 (14.4)	97.0 (12.5)	95.2 (14.3)	−5.2 (−6.9 to −3.6)	<0.0001
Fat mass, kg (DEXA)	41.9 (11.0)	–	38.0 (10.1)	41.9 (10.4)	–	41.8 (10.8)	−3.9 (−5.3 to −2.5)	<0.0001
Fat percentage, %kg (DEXA)	44.5 (5.5)	–	42.7 (5.6)	43.4 (6.8)	–	43.2 (6.9)	−2.1 (−3.0 to −1.1)	<0.0001
Lean mass, kg (DEXA)	47.0 (43.0–51.9)	–	46.0 (41.2–52.7)	49.8 (44.6–53.6)	–	48.9 (45.1–53.2)	−0.7 (−1.5 to 0.1)	0.08
ASMM, kg (DEXA)	21.5 (19.1–24.3)	–	21.1 (18.6–24.7)	22.0 (20.6–24.5)	–	22.2 (20.7–24.7)	−0.5 (−1.0 to 0.1)	0.09
Waist circumference, cm	109 (14)	104 (13)	101 (11)	112 (13)	109 (8)	111 (12)	−6 (−9 to −4)	<0.0001
Females (*n* ​= ​55)	108 (14)	103 (13)	100 (10)	111 (14)	108 (8)	110 (13)	−6 (−9 to −4)	<0.0001
Males (*n* ​= ​10)	117 (8)	113 (10)	110 (12)	116 (9)	116 (4)	115 (10)	−5 (−9 to −2)	0.02
Hand grip strength, kg	28 (24–32)	–	27 (24–32)	26 (23–32)	–	27 (23–32)	1 (−2 to 4)	0.5
Bone mineral density, g/cm^2^	1.20 (0.15)	–	1.19 (0.15)	1.22 (0.15)	–	1.22 (0.14)	−0.01 (−0.03 to 0.01)	0.3
**Metabolic markers**								
Insulin, pmol/l∗	54 (45–74)	38 (34–59)	42 (31–65)	68 (49–88)	59 (49–85)	58 (42–87)	–	0.01
ALAT∗	25.5 (18.5–34.5)	24.5 (17.8–34.3)	23.5 (16.8–27.3)	26.0 (19.8–35.0)	24.5 (20.0–39.5)	26.0 (19.8–38.3)	–	0.03
ASAT	22.5 (20.0–26.3)	24.0 (20.5–27.0)	23.0 (20.0–26.0)	24.5 (20.0–29.3)	24.0 (23.0–27.8)	23.0 (21.8–30.3)	−2.0 (−5.3 to 1.3)	0.2
eGFR	82 (77–90)	88 (82–90)	88 (82–90)	87 (74–90)	87 (65–90)	88 (76–90)	2.8 (0.1–5.5)	<0.05

Outcomes for the total group (n ​= ​64), results reported as mean (SD) when normally distributed and median (Q1–Q3) when skewed. P-values are based on a linear regression (DEXA outcomes and hand grip strength) or linear mixed model with random effect (all other outcomes) for between group analysis, adjusted for baseline values. If model assumptions were not met (∗) a log transformation was applied and the between group difference is not available. Additional adjustment for covariates (sex, age, and BMI) did not change outcomes, whereby weight, fat mass, BMI, waist circumference, lean mass, and ASMM were not adjusted for BMI. DEXA ​= ​Dual-energy X-ray absorptiometry, ASMM ​= ​Appendicular skeletal muscle mass, ALAT ​= ​Alanine aminotransferase, ASAT ​= ​aspartaat aminotransferase, eGFR ​= ​estimated glomerular filtration rate.

All MSOA subgroups showed significant improvements in body weight and fatmass. Participants with only hip or knee and hip MSOA had greater reductions in weight and fat mass as compared to the whole MSOA cohort and knee MSOA subgroup (Supplementary Table 2). There was no change in lean and appendicular skeletal muscle mass in participants with only knee MSOA and both knee and hip MSOA. However, those with only hip MSOA showed a reduction in lean and appendicular skeletal muscle mass, the later being statistically significant (Supplementary Table 2).

### 3.3 Other outcomes

As previously published, CRP, fasting glucose, HbA1c, and LDL cholesterol decreased significantly in the intervention group compared to controls [2]. In this secondary analysis the PFJ group also showed significantly reduced insulin and ALAT levels, along with a significant improvement in eGFR, all compared to controls (Table 1).

### 3.4 MRS spectroscopy

Thirty-two MSOA participants underwent an MRI scan at the start and end of the PFJ RCT, *n* ​= ​17 from the PFJ group and *n* ​= ​15 from the control group (Fig. 2). In this subgroup 84% were female with a mean age (63 years (SD 7)), BMI (34 ​kg/m2 (SD 5)), and fat mass (42 ​kg (SD 10)) (Supplementary Table 2), similar to the entire MSOA group. Two dropouts occurred in each group before the second MRI, and some spectra were excluded due to interpretability issues (PFJ group: liver *n* ​= ​1, VAT *n* ​= ​2; Control group: liver *n* ​= ​2, VAT *n* ​= ​3).

**Fig. 3:**
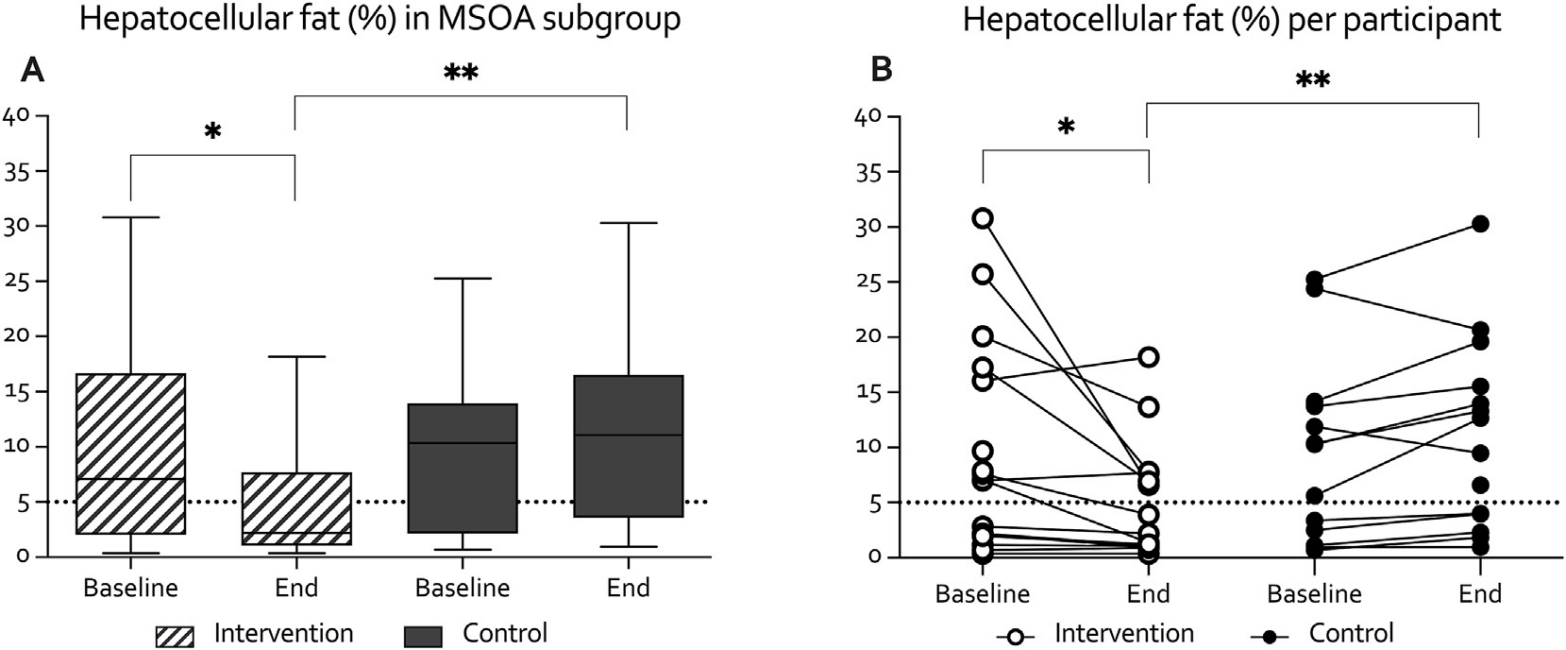
Hepatocellular fat from MR spectroscopy per trial arm (A) and hepatocellular fat changes for individual participants (B) for the MSOA subgroup who underwent an MRI scan (*n* ​= ​32). Box plots show median (IQR) with error bars (5–95 percentile). A linear regression analysis was used to compare the intervention and control groups after 16 weeks (controlled for baseline values). Additional adjustment for covariates (sex, age, and BMI) did not change outcomes. Differences between groups at baseline and within groups were analyzed respectively with a two or one-sample *t*-test when normally distributed or a Wilcoxon-Rank test when skewed. The dotted line in A shows the cut-off for NAFLD. P-value: ∗∗∗<0.001, ∗∗<0.01, ∗<0.05.

After 16 weeks, the PFJ group showed reduced hepatocellular fat (−6.5% CI 95%−9.9 to −3.0) compared to controls (Fig. 3). No significant differences were observed in (poly)unsaturated fatty acids to saturated fat ratios in VAT between the groups (Table 2).

**Table 2:** MR spectroscopy outcomes in a subgroup of metabolic syndrome-associated osteoarthritis participants.

Characteristic	Plants for Joints group (*n* ​= ​17)		Control group (*n* ​= ​15)		Difference between	*p*-value
	baseline	16 weeks	baseline	16 weeks	groups (95 ​% CI)	
**Liver**						
Liver fat fraction, %	7.1 (2.1–16.1)	2.2 (1.1–7.3)	10.4 (2.7–13.9)	11.1 (4.0–15.2)	−6.5 (−9.9 to −3.0)	0.001
**Visceral adipose tissue**						
α-olefin to methylene ratio	0.23 (0.03)	0.23 (0.03)	0.21 (0.05)	0.24 (0.02)	−0.01 (−0.03 to 0.01)	0.2
Olefin to methylene ratio	0.10 (0.02)	0.11 (0.02)	0.11 (0.03)	0.10 (0.01)	0.01 (0.00–0.02)	0.07
α-olefin ​+ ​olefin to methylene ratio	0.33 (0.04)	0.34 (0.04)	0.32 (0.07)	0.34 (0.03)	0.00 (−0.03 to 0.03)	1.0
Diacyl to methylene ratio	0.01 (0.01)	0.01 (0.01)	0.01 (0.01)	0.01 (0.01)	0.00 (−0.01 to 0.00)	0.5
α-olefin ​+ ​olefin ​+ ​diacyl to methylene ratio	0.33 (0.05)	035 (0.04)	0.33 (0.08)	0.35 (0.04)	0.00 (−0.03 to 0.03)	0.8

Outcomes for the MSOA subgroup who underwent an MRI scan (*n* ​= ​32), reported as mean (SD) when normally distributed and median (Q1–Q3) when skewed. Amplitude ratios were calculated for the (poly)unsaturated fat peaks (olefin (unsaturated fat), α-olefin (unsaturated fat), and/or diacyl (poly unsaturated fat)) divided by the saturated fat (methylene) peak. P-values are based on a linear regression for between group analysis, adjusted for baseline values. Additional adjustment for covariates (sex, age, and BMI) did not change outcomes.

Clinical outcome changes in this subgroup mostly mirrored those of the total MSOA group, except for insulin and ALAT levels, which decreased significantly within the MRS intervention group compared to baseline but were not significantly different from the control group post-intervention (*p* ​= ​0.05 and 0.08 respectively; Supplementary Table 3). Due to the small sample size additional subgroup analyses by MSOA location were not performed.

## 4 Discussion

The 16-week Plants for Joints randomized controlled trial resulted in a significant improvement of pain, stiffness, and physical function in people with metabolic syndrome-associated knee and/or hip osteoarthritis as compared to usual care [2]. This secondary analysis of the PFJ trial further studied changes in body composition, showing sustained muscle mass, strength, and BMD despite significant reductions of weight and fat mass. Furthermore, the intervention led to a significant reduction in liver fat alongside improved ALAT levels, although VAT composition did not change.

Weight loss is recommended as an important treatment approach for people with knee and hip OA [25]. Yet, often fat loss coincides with loss of lean mass, of particular relevance for populations with metabolic disease given the importance of skeletal muscle for metabolic health [26]. To combat loss of lean mass weight loss interventions often focus on higher intake of dietary protein and intensive muscle strengthening exercises [26]. In the PFJ study, despite significant weight and fat mass reductions and decreased protein intake (from 0.91 to 0.79 ​g/kg body weight adjusted to match a BMI of 27.5 for those with BMI ≥30) [2], lean mass and appendicular skeletal muscle mass were unchanged. This aligns with previous studies showing low-fat (plant-based) diets better preserve lean mass compared to low-carbohydrate (animal-based) diets in overweight individuals, despite lower protein intake in the low-fat group [26,27]. Yet, one-year after the PFJ intervention a small yet significant reduction in lean mass (−0.8 ​kg 95% CI –1.3 to −0.4) and appendicular skeletal muscle mass (−0.7 ​kg 95% CI –1.0 to −0.4) was observed within the MSOA group [28]. Therefore, while a plant-based diet combined with exercise can effectively aid weight loss while preserving muscle mass and strength, special attention to strength training and sufficient protein intake, potentially with supplementation, is necessary, especially in older adults and those with chronic inflammatory conditions [7,[29], [30], [31], [32]].

Differences are present in the pathophysiology, anatomy, and biomechanics of knee and hip OA [33]. While anatomical and biomechanical differences may lead to a different expression of OA in different joints such as hip and knee, in the present study subjects were selected for a predominantly metabolic origin of their OA with the requirement of metabolic syndrome. In that sense, knee and hip are similar in that they both have intrasynovially located fat pads that are metabolically active and contribute to the osteoarthritic inflammatory process. In this study we found significant improvements in body weight and fat mass in all MSOA subgroups. While there were some differences in baseline weight, fat mass, and lean mass, and between group differences found at the end of the trial, caution is needed when interpreting these findings due to small sample sizes.

In this study there was no significant change in BMD in those following the PFJ intervention, in contrast with previous studies associating a lower BMD and higher fracture risk with vegan diets, possibly due to lower amounts of certain nutrients [9]. Yet, in a properly planned vegan diet, like the PFJ intervention, these nutrient deficiencies should not occur [34]. In fact, when comparing a high-quality vegan diet to other diets, no difference in BMD was found [35]. On the other hand, four months could be considered too short to detect a change in bone mineral density [36]. An additional DEXA performed one year post-PFJ intervention showed a significant decrease in BMD compared to baseline (mean bone density 1.21 to 1.19 ​g/cm^2^, within group difference −0.02 (95% CI –0.03 to −0.01)), although the T-score remained within the normal range (mean T-score 1.09 to 0.92, within group difference −0.18 (−0.32 to −0.03)) [28]. Yet, the 1.7% reduction in BMD observed one-year post-intervention is similar to the loss of bone-mineral density typically seen in women ages 30–94 (median age 60; −1%) and post-menopausal women (−1.9%) [37,38].

This secondary analysis found significant improvements in insulin concentration, liver fat fraction, and ALAT levels. While insulin remained within the reference interval (12–96 ​pmol/L (Amsterdam UMC)) and is not of clinical significance on its own, alongside reductions in fasting blood glucose and HbA1c, the decrease supports the intervention's effect on reducing insulin resistance. Furthermore, liver fat fraction decreased significantly from 7.1 to 2.2% within the intervention group, below the NAFLD threshold (≥5%), indicating a clinically relevant reduction in liver steatosis [39]. Although ALAT levels remained within the normal range, it has been shown to be an independent predictor of NAFLD, with a step-wise increase in NAFLD incidence as ALAT rises, even within normal limits [40]. Overall, these findings highlight the PFJ intervention's impact on improving insulin resistance and liver steatosis, and tie together the central role of hepatocellular lipids in insulin resistance [10].

These findings are supported by previous findings associating body and liver fat, insulin resistance, and inflammation [10]. Specifically, Kahleova et al. showed a low-fat vegan diet significantly reduced body weight, fat mass, and hepatocellular fat (−1.2%) in adults with a BMI of 28–40 compared to controls [41]. Whole-food plant-centered diets as well as specific food groups such as vegetables, fruits, whole grains, and nuts are also associated with a lower risk of NAFLD [[41], [42], [43], [44], [45]], likely due to weight loss, reduced inflammation, and improved insulin resistance attributed to the abundance of polyphenols, antioxidants, and fiber [42]. Although exercise alone can reduce hepatocellular fat, ALAT, and aspartate aminotransferase levels [46,47], combined dietary and exercise interventions are more effective at improving NAFLD [48].

In the MRS subgroup, no significant differences in VAT composition were observed between or within the trial arms. These findings were unexpected as VAT composition can change based on dietary intake of poly- and monounsaturated fat and saturated fat [14], and participants significantly reduced saturated fat intake [2]. This result could be attributed to the short duration of the intervention, as adipose tissue turnover typically takes six to nine months [14]. Additionally, the small sample size may have limited the statistical power to detect significant changes. To date few studies have used MRS to quantify VAT composition [23,24]. While gas chromatography using tissue biopsies is the gold standard for determining fatty acid composition, MRS is a non-invasive technique [23]. Further studies are needed to assess reproducibility of this technique and changes in VAT over longer periods of time after dietary interventions.

Limitations of this study include the inability to determine the individual impact of the lifestyle factors on the results, due to the multidisciplinary approach. Also, due to the limited study period and small sample size, some effects were potentially not detected. Moreover, the power calculation for this study was not targeted at the secondary outcomes. Lastly, since participants were selected based on a metabolic origin of OA, individuals with MSOA may be more responsive to interventions like PFJ compared to other OA phenotypes. This limits the generalizability of the findings to broader OA populations.

## 5 Conclusion

The PFJ intervention appears to preserve muscle mass, strength, and BMD while significantly reducing weight, fat mass, and liver fat in people with MSOA. These results highlight the intervention's broader impact on metabolic health and body composition beyond treating symptoms alone.

## Credit author statement

**C.A. Wagenaar**: Methodology, Validation, Investigation, Data Curation, Writing - Original Draft, Visualization, Project administration. **W. Walrabenstein**: Conceptualization, Methodology, Investigation, Data Curation, Writing - Original Draft, Project administration, Funding acquisition. **C.S. de Jonge**: Methodology, Validation, Writing - Original Draft, Writing - Review & Editing. **M. Bisschops**: Validation, Formal analysis, Data Curation, Writing - Review & Editing. **M. van der Leeden**: Methodology, Writing - Review & Editing. **M. van der Esch**: Methodology, Writing - Review & Editing. **P.J.M. Weijs**: Methodology, Writing - Review & Editing. **M.A. Troelstra**: Methodology, Writing - Review & Editing. **M.A. Korteweg**: Methodology, Writing - Review & Editing. **A.J. Nederveen**: Methodology, Supervision, Resources, Writing - Review & Editing. **D. van Schaardenburg**: Conceptualization, Methodology, Supervision, Funding acquisition, Resources, Writing - Review & Editing.

## Role of the funding source

The RCT is funded by Reade (Amsterdam, the Netherlands), Reade Foundation (Amsterdam, the Netherlands), Stichting Vermeer 14 (private foundation, Amsterdam, the Netherlands) and W.M. de Hoop Stichting (private foundation, Bussum, the Netherlands). The position of CAW is funded by The Netherlands Organisation for Health Research and Development (ZonMw) no. 555003210. The funders had no role in the design and conduct of the study; collection, management, analysis, and interpretation of the data; preparation, review, or approval of the manuscript; and decision to submit the manuscript for publication.

## Availability of data and materials

The datasets during and/or analyzed during the current study available from the corresponding author on reasonable request.

## Declaration of generative AI and AI-assisted technologies in the writing process

During the preparation of this work the authors used ChatGPT in order to improve readability and language. After using this tool, the authors reviewed and edited the content as needed and take full responsibility for the content of the publication.

## Declaration of competing interest

Authors CAW, WW, and DvS hold shares in Plants for Health, a limited liability company, which aims to have a positive impact on society and the environment and provide an adapted version of the Plants for Joints program as an additional treatment option for people with rheumatic conditions. All other authors report no conflict of interest.
