# Corrigendum to ‘Effect of a multidisciplinary lifestyle intervention on body composition in people with osteoarthritis: Secondary analysis of the “Plants for Joints” randomized controlled trial’ [Osteoarthritis and Cartilage Open 6 (2024) 100524]

**DOI:** 10.1016/j.ocarto.2025.100722

**Published:** 2025-12-07

**Authors:** C.A. Wagenaar, W. Walrabenstein, C.S. de Jonge, M. Bisschops, M. van der Leeden, M. van der Esch, P.J.M. Weijs, M.A. Troelstra, M.A. Korteweg, A.J. Nederveen, D. van Schaardenburg

**Affiliations:** a Reade Center for Rheumatology and Rehabilitation, Amsterdam, the Netherlands; b Amsterdam UMC Location University of Amsterdam, Department of Clinical Immunology and Rheumatology, Meibergdreef 9, Amsterdam, the Netherlands; c Amsterdam Rheumatology and Immunology Center, Amsterdam, the Netherlands; d Amsterdam UMC Location University of Amsterdam, Department of Radiology and Nuclear Medicine, Meibergdreef 9, Amsterdam, the Netherlands; e Amsterdam Gastroenterology Endocrinology Metabolism Research Institute, Amsterdam, the Netherlands; f Amsterdam UMC Location Vrije Universiteit Amsterdam, Department of Rehabilitation Medicine, Boelelaan 1117, Amsterdam, the Netherlands; g Amsterdam Movement Sciences Research Institute, Amsterdam, the Netherlands; h Center of Expertise Urban Vitality, Amsterdam University of Applied Sciences, Faculty of Health, Amsterdam, the Netherlands; i Department of Nutrition and Dietetics, Center of Expertise Urban Vitality, Amsterdam University of Applied Sciences, Amsterdam, the Netherlands; j Amsterdam UMC Location Vrije Universiteit Amsterdam, Department of Nutrition and Dietetics, Boelelaan 1117, Amsterdam, the Netherlands

The authors regret that there is an error in the abstract of the article. Specifically, in the Results section, it currently states: “In the subgroup who underwent MRI scans, the PFJ group had a greater reduction in HCL (−6.5 ​%; 95 ​% CI −9.9, 3.0) compared to controls, with no observed differences in VAT composition.” However, the upper bound of the 95 ​% confidence interval should read −3.0, not 3.0, as is correctly reflected in the main text, and indicates a statistically significant reduction in HCL. The authors would like to apologise for any inconvenience caused.

