# Supplementary material for: Effect of a multidisciplinary lifestyle intervention on body composition in people with osteoarthritis: Secondary analysis of the “Plants for Joints” randomized controlled trial
Source: Osteoarthr Cartil Open. 2024 Oct 1;6(4):100524. doi: 10.1016/j.ocarto.2024.100524 (PMC11491948; doi:10.1016/j.ocarto.2024.100524)
Supplement: Multimedia component 1 [file mmc1.pdf]

Supplementary Table 1. MR spectroscopy visceral adipose tissue prior knowledge

Peak	Chemical shift (ppm)	Type	Amplitude	Relative phase	Line width (ppm)	Frequency (ppm)	Shape
**1**	5.29	Olefin	estimated	fixed - 0.0	soft constraints – 0.1 – 3.5	soft constraints - 5.25 - 5.35	fixed - Gaussian
**2**	5.19	Glycerol	fixed ratio - peak #3*0.5	fixed - 0.0	soft constraints - 0.1 – 3.5	soft constraints - 5.15 - 5.25	fixed - Gaussian
**3**	4.3	Glycerol	estimated	fixed - 0.0	soft constraints - 0.1 – 3.5	fixed shift - peak #4 + 25.5	fixed - Gaussian
**4**	4.09	Glycerol	fixed ratio - peak #3*1.0	fixed - 0.0	soft constraints - 0.1 – 3.5	soft constraints - 4.0 - 4.2	fixed - Gaussian
**5**	2.74	Diacyl	estimated	fixed - 0.0	soft constraints - 0.1 – 3.5	soft constraints - 2.7 - 2.8	fixed - Gaussian
**6**	2.24	α-Carboxyl	estimated	fixed - 0.0	soft constraints - 0.1 – 3.5	soft constraints - 2.19 - 2.29	fixed - Gaussian
**7**	2.02	α-Olefin	estimated	fixed - 0.0	soft constraints - 0.1 – 3.5	soft constraints - 1.97 - 2.07	fixed - Gaussian
**8**	1.6	β-Carboxyl	estimated	fixed - 0.0	soft constraints - 0.1 – 3.5	soft constraints - 1.5 - 1.7	fixed - Gaussian
**9**	1.3	Methylene	estimated	fixed - 0.0	soft constraints - 0.1 – 3.5	soft constraints - 1.25 - 1.35	fixed - Gaussian
**10**	0.9	Methyl	estimated	fixed - 0.0	soft constraints - 0.1 – 3.5	soft constraints - 0.4 - 1.04	fixed - Gaussian

Chemical shift for the peaks based on Peterson et al., 2020 and Hamilton et al., 2017.

Supplementary Table 2. Body composition outcomes for metabolic syndrome-associated osteoarthritis subgroups

	All OA participants (*n* = 64)		Knee OA (*n* = 25)		Hip OA (*n* = 12)		Knee and hip OA (*n* = 27)	
**Characteristic**	**Between group difference (95% CI)**	***p*-value**	**Between group difference (95% CI)**	***p*-value**	**Between group difference (95% CI)**	***p*-value**	**Between group difference (95% CI)**	***p*-value**
Weight, kg	–5.2 (–6.9 to –3.6)	<0.0001	–4.0 (–6.3 to –1.7)	0.003	–6.1 (–10.1 to –2.0)	0.02	–6.1 (–8.7 to –3.5)	0.0001
Fat mass, kg (DEXA)	–3.9 (–5.3 to –2.5)	<0.0001	–2.7 (–4.7 to –0.7)	0.01	–5.4 (–8.7 to –2.0)	0.01	–4.2 (–6.5 to –1.8)	0.002
Lean mass, kg (DEXA)	–0.7 (–1.5 to 0.1)	0.08	–0.3 (–1.6 to 1.0)	0.7	–2.0 (–3.8 to 0.2)	0.06	–0.8 (–2.3 to 0.7)	0.3
ASMM, kg (DEXA)	–0.5 (–1.0 to 0.1)	0.09	0.1 (–0.8 to 0.9)	0.9	–1.2 (–2.0 to –0.4)	0.02	–0.6 (–1.5 to 0.4)	0.3
Waist circumference, cm	–6 (–9 to –4)	<0.0001	–5 (–9 to –1)	0.04	–6 (–11 to –2)	0.02	–7 (–10 to –3)	0.001
Bone mineral density, g/cm2 (DEXA)	–0.01 (–0.03 to 0.01)	0.3	–0.03 (–0.06 to 0.01)	0.2	–0.02 (–0.02 to 0.06)	0.3	–0.01 (–0.04 to 0.01)	0.3

Outcomes for the total group (*n* = 64), and subgroups of knee OA (*n* = 25), hip OA (*n* = 12), and those with both knee and hip OA (*n* = 27). P-values are based on a linear regression (fat mass, lean mass, ASMM, and bone mineral density) or linear mixed model with random effect (weight and waist circumference) for between group analysis (intervention vs. control group) at the end of the 16-week Plants for Joints intervention, adjusted for baseline values. Additional adjustment for covariates (sex, age, and BMI) did not change outcomes, whereby only bone mineral density was adjusted for BMI. DEXA = Dual-energy X-ray absorptiometry, ASMM = Appendicular skeletal muscle mass.

Supplementary Table 3. MRS subgroup clinical outcomes

	Plants for Joints group (*n* = 17)		Control group (*n* = 15)		Difference between	
**Characteristic**	**baseline**	**16 weeks**	**baseline**	**16 weeks**	**groups (95% CI)**	***p–value***
**Body composition**						
Weight, kg	94.1 (14.3)	87.7 (13.8)	94.6 (16.4)	94.5 (16.6)	–4.2 (–6.4 to –2.1)	<0.001
Body mass index, kg/m2	32.3 (3.8)	30.0 (3.5)	34.4 (5.3)	34.4 (5.3)	–1.5 (–2.3 to –0.8)	<0.001
Fat mass, kg (DEXA)	40.8 (9.1)	35.8 (8.4)	42.5 (10.4)	42.9 (10.7)	–5.5 (–7.7 to –3.4)	<0.0001
Waist circumference, cm	109 (10)	101 (9)	112 (16)	111 (13)	–7 (–10 to –4)	<0.001
**Inflammation**						
C-reactive protein, mg/l	2.4 (1.1 2.2)	1.3 (0.9–2.4)	2.6 (1.4–3.8)	3.0 (1.3–6.1)	–1.0 (–2.0 to –0.1)	0.04
**Metabolic markers**						
Fasting blood glucose, mmol/l	5.8 (5.4–6.2)	5.3 (5.1–5.5)	5.7 (5.6–6.5)	5.8 (5.2–6.5)	–0.5 (–0.8 to –0.1)	0.01
HbA1c, mmol/mol	39 (4)	37 (4)	42 (7)	42 (7)	–2 (–3 to –1.0)	0.005
Insulin, pmol/l	49 (40–91)	33 (21–78)	68 (46–84)	61 (40–76)	–	0.05
Systolic blood pressure, mmhg	144 (15)	144 (15)	152 (21)	146 (20)	–1 (–10 to 9)	0.9
Diastolic blood pressure, mmhg	92 (8)	88 (9)	95 (9)	88 (11)	0 (–5 to 5)	1.0
LDL-cholesterol, mmol/l	3.3 (3.0–4.4)	3.1 (2.2–4.2)	3.8 (2.7–4.8)	3.6 (2.9–4.8)	–0.4 (–0.8 to –0.1)	0.02
HDL-cholesterol, mmol/l	1.61 (0.38)	1.47 (0.32)	1.54 (0.53)	1.40 (0.57)	–0.0 (–0.2 to 0.2)	0.9
Triglycerides, mmol/l	1.4 (1.2–1.6)	1.6 (1.0–1.9)	1.4 (1.1–1.9)	1.6 (1.3–2.8)	–0.2 (–0.4 to 0.1)	0.3
ALAT, IU/l	31.0 (27.0–37.0)	25.0 (21.0–27.0)	27.0 (16.5–36.0)	25.0 (18.5–39.0)	–	0.08
ASAT, IU/l	26.0 (21.0–28.0)	24.5 (22.0–27.3)	28.0 (21.0–32.5)	24.0 (22.0–32.5)	–2.7 (–8.8 to 3.4)	0.4

Outcomes for the OA subgroup who underwent an MRI scan (*n* = 32), descriptives and results reported as mean (SD) when normally distributed and median (Q1 – Q3) when skewed. P-values are based on a linear regression (MR spectroscopy and DEXA outcomes) or linear mixed model with random effect (all other outcomes) for between group analysis, adjusted for baseline values. As model assumptions were not met for insulin, a log transformation was applied and the between group difference is not available. Additional adjustment for covariates (sex, age, and BMI) did not change outcomes, except for insulin (*p* = 0.02). Weight, fat mass, BMI, and waist circumference were not adjusted for BMI.
